# A Case of Hemodynamically Unstable Sick Sinus Syndrome Secondary to Refeeding Syndrome

**DOI:** 10.7759/cureus.14625

**Published:** 2021-04-22

**Authors:** Michael Mgerian, Suzanne Havican, Frances Wolf, Nioti Karim

**Affiliations:** 1 Family Medicine / Graduate Medical Education (GME) Department, HCA Houston Healthcare West, Houston, USA; 2 Internal Medicine, HCA Houston Healthcare West, Houston, USA

**Keywords:** refeeding syndrome, sick sinus syndrome, cardiogenic shock, cardiac arrhythmia, tachy-brady syndrome

## Abstract

Prolonged malnourished states can predispose patients to refeeding syndrome with the uncommon complication of cardiogenic shock if not corrected initially. While refeeding syndrome is well studied its complications may not be easily identified in the setting of rapid deterioration. This case report reviews the events of a 57-year-old male who was brought in by law enforcement, for altered mental status and agitation, after being found wandering in the woods. The patient was initially hemodynamically stable but developed cardiogenic shock from sick sinus syndrome. The patient's shock was non-responsive to IV fluid resuscitation and required ICU admission. Congestive heart failure, ischemic cardiomyopathy, substance abuse, myocarditis, and endocarditis were considered but ruled out. Patient's symptoms improved after electrolyte repletion following brief ICU admission with dopamine drip.

## Introduction

Refeeding syndrome (RFS) can result in a progressive and rapid deterioration of a patient's medical state if not monitored carefully. Cardiac myocytes are particularly sensitive to RFS; transient myocyte dysfunction can progress to sick sinus syndrome with bradycardia. This is largely mediated due to electrolyte abnormalities secondary to deprivation of intake and exacerbated by insulin release and impaired cell respiration from thiamine deficiency. Patients who are malnourished need to be more closely monitored for electrolyte abnormalities and re-introduced to intake of macronutrients through a graded and closely monitored medical process [1].

While RFS may include rhabdomyolysis, hemolysis, delirium, encephalopathy, pulmonary edema, our case report aims to focus on the cardiogenic aspect. Cardiogenic decompensation is the leading cause of death in RFS and may also be an early sign [1].

## Case presentation

A 57-year-old male with an unknown past medical history presented to our emergency department by law enforcement and emergency medical services (EMS) after being found wandering in the woods. Reportedly the patient has been neglecting to eat or drink with the intent of starving himself to death. It was estimated that he had been depriving himself of food for eight to 10 days. On arrival and admission, the patient was acutely agitated, non-cooperative, and psychotic. There was unclear history of prior alcohol or drug abuse. During admission, the patient had the following vitals: a heart rate of 78 beats per minute, respiratory rate of 16 breaths per minute, blood pressure 127/40 mmHg, pulse oximetry 98 on room air, an axillary temperature of 96.2°F. Initial cardiac telemetry showed normal sinus rhythm. Initial electrolytes values were as seen in Table 1, column ‘Admission’.

**Table 1:** Comparison of medical lab values between admission and hospital day 2 BUN: Blood urea nitrogen; AST: Aspartate transaminase; ALT: Alanine transaminase; TSH: Thyroid-stimulating hormone; TNP: Test not performed.

Name of assay	Admission	Hospital Day 2	Reference Range
Na	155 mmol/L	148 mmol/L	135 - 145 mmol/L
K	4.0 mmol/L	3.4 mmol/L	3.6 - 5.2 mmol/L
Cl	109 mmol/L	113 mmol/L	96 - 106 mmol/L
HCO3	25 mmol/L	29 mmol/L	22 - 29 mmol/L
Creatinine	1.40 mg/dl	0.80 mg/dl	0.84 - 1.24 mg/dl
BUN	35 mg/dl	16 mg/dl	7.0 - 20 mg/dl
Glucose	126 mg/dl	125 mg/dl	70 - 130 mg/dl (fasting state)
Ca	10.6 mg/dl	8.3 mg/dl	8.6 - 10.3 mg/dl
Corrected Ca	9.6md/dl	TNP	
Mg	3.3 mg/dl	2.5 mg/dl	1.7 - 2.2 mg/dl
Phosphorus	5.1 mg/dl	2.3 mg/dl	3.4 - 4.5 mg/dl
Albumin	5.2 g/dl	TNP	3.5 - 5.0 g/dl
AST	5.2 U/L	TNP	5.0 - 40 units/L
ALT	21 U/L	TNP	7 - 55 units/L
Total Protein	8.2 g/L	TNP	6.0 - 8.3 g/DL
Total Bilirubin	3.0 g/L	TNP	0.1 - 1.2 g/DL
TSH	TNP	1.650	0.45 - 4.68 MIU/L

The patient refused intravenous (IV) and per oral (PO) medications including IV fluids. The patient had an unknown history of alcohol abuse and was started on IV fluids with supplementation of IV thiamine/multivitamin “Banana Bag” in the emergency department but the infusion was not able to be completed as the patient removed his own IV access. Following admission, the patient was allowed to eat an unrestricted diet.

On hospital day 2, the patient continued to be delirious, uncooperative, and agitated. His blood pressure was gradually declining from an early morning reading of 105/70 to 65/40 by noon; his heart rate on cardiac telemetry demonstrated a high degree of variance with a range of 50 to 140 beats per minute.

**Figure 1:**
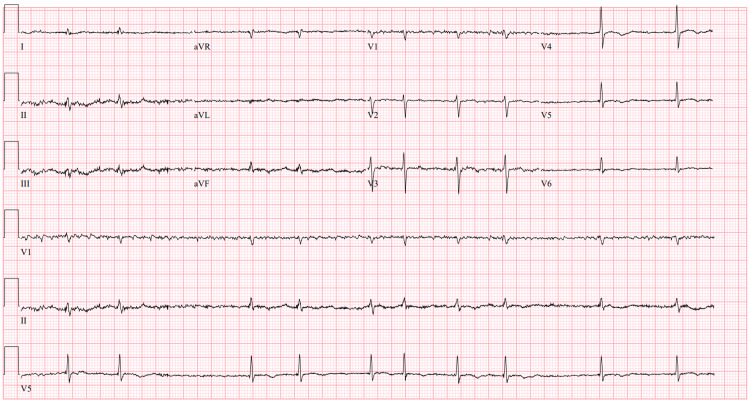
Tachycardia with sinus arrest

**Figure 2:**
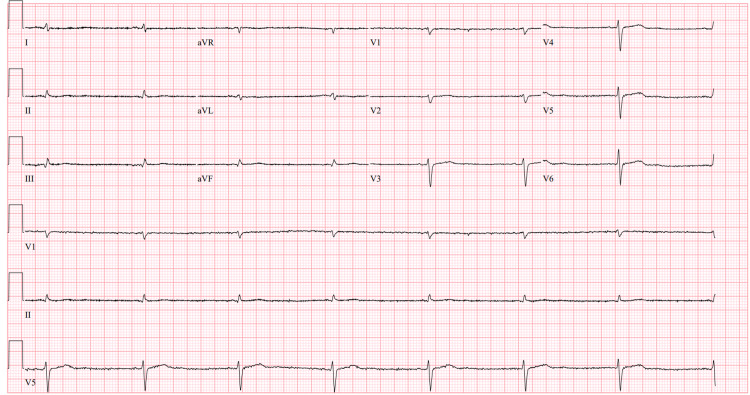
Persistent sinus bradycardia

A transthoracic echocardiogram was ordered showing ejection fraction 50-54%, no significant diastolic dysfunction or wall motion abnormality, and trivial valvulopathy. A review of cardiac telemetry readings showed a pattern suggestive of sick sinus syndrome with tachycardia and sinus arrest (Figure 1), followed by progression into persistent bradycardia with a mean heart rate of 40-45 (Figure 2). Troponin-I levels were normal.

The patient was lethargic but otherwise not in acute distress. Crystalloid and colloid fluids included isotonic solutions of Plasmalyte and Lactated Ringers and 50 grams of 5% albumin solution; with a total volume of 3 liters. Despite volume resuscitation, the patient remained hypotensive. Systolic blood pressure continued to trend down to 55 mmHg. A repeat of electrolyte labs before IV fluid resuscitation revealed values as stated in Table 1, column “Hospital day 2”.

The patient was admitted to ICU and placed on a dopamine drip to mitigate the progression of cardiogenic shock. He was monitored with serial electrolyte labs and free feeds discontinued to prevent worsening of the electrolyte abnormalities. Upon normalization of the patient’s electrolytes, he was taken off the dopamine drip once his severe bradycardia had resolved. The patient’s sick sinus syndrome resolved and he was able to be downgraded from the ICU after two more days.

## Discussion

Sick sinus syndrome is a well-known complication of refeeding syndrome, predominantly mediated through serum electrolyte abnormalities causing abnormal conduction of cardiac signals [2]. Depletion of phosphorus causes a reduction in ventricular myocyte contractility further propagating cardiogenic shock. Phosphorus depletion is further exacerbated by increasing demand for adenosine triphosphate (ATP) production after feeds are resumed; the rapid increase in phosphorus causes further serum depletion of the electrolyte resulting in abnormal cardiac myocyte activity and signal conduction [3,4]. In RFS hypokalemia is caused by K influx into the intracellular compartment via insulin-mediated Na/K+ exchange pumps; additionally, patients are K-depleted due to their malnourished state [5]. Hypomagnesemia further exacerbates hypokalemia by preventing potassium reuptake by nephrons [6]. Thiamine deficiency plays a critical role in ATP metabolism in the citric acid cycle (CAC) and pyruvate and lactic acid metabolism. Furthermore, thiamine deficiency is well known for causing cardiac contractility failure [7]. The American Society of Parenteral and Enteral Nutrition (ASPEN) has published revised guidelines for the identification, prevention, and management of RFS [8,9]. Management guidelines include repletion of lost fluid volume with isotonic fluids, repletion of electrolytes, thiamine, and followed by a gradual progressive graded reintroduction of calories starting at 10 kcal/kg/day and gradually increased to full feeds over 4-7 days. In more extreme cases (BMI < 14) a starting value of 5 kcal/kg/day is recommended. Additionally, critical patients should have close serial monitoring of electrolytes and repletion. Further recommendations are made in supplementing phosphorus at a rate of 10-15 mmol per 1,000 kcal of feeds provided. Though there is some discrepancy as to the precise rate at which refeeding should be initiated, the overwhelming consensus cautions against exceeding 20 kcal/kg/day upon starting and no more than 400 of additional kcal per day. If sick sinus syndrome with persistent bradycardia develops, dopamine drips can be used in an ICU setting to help reverse cardiogenic shock. The chronotropic and inotropic nature of dopamine aids in both the bradycardia and reduced contractility of cardiogenic shock associated with RFS [9,10].

In our patient, compliance was a major issue in following proper practice in RFS treatment, prevention, and management. With the patient refusing labs and being combative with medical team members when obtaining necessary labs and administration of IV medications, gaps in monitoring and repletion of electrolytes temporarily occurred. Additionally, he was allowed to eat without restriction due to his refusal of IV therapy and removal of IV access.

## Conclusions

This case highlights the importance of early identification and proper correction of refeeding syndrome. While refeeding syndrome and its complications are well-known it can be difficult to identify initially. Unfortunately, cardiogenic shock and subsequent death is often both an early and terminal presentation of such a case. While RFS may have additional manifestations namely rhabdomyolysis, encephalopathy, seizures, and hemolysis, they may not be apparent or present during the early phase.

Failure to identify and correct for refeeding syndrome can result in hemodynamic instability with cardiogenic shock that may mimic sick sinus syndrome. Though the sick sinus may be transient it will often require an ICU admission with dopamine infusion to assist with the correction of bradycardia. Patients with a high index of suspicion of being in a prolonged malnourished state should undergo a progressively graded calorie-controlled diet with close monitoring of electrolyte labs to account for correction.
